# Prostate cancer detection using multiparametric 3 – tesla MRI and fusion biopsy: preliminary results

**DOI:** 10.1590/S1677-5538.IBJU.2015.0204

**Published:** 2016

**Authors:** Thais Caldara Mussi, Rodrigo Gobbo Garcia, Marcos Roberto Gomes de Queiroz, Gustavo Caserta Lemos, Ronaldo Hueb Baroni

**Affiliations:** 1 Departamento de Radiologia e Diagnóstico por Imagem do Hospital Israelita Albert Einstein, São Paulo, SP, Brasil; 2 Departamento de Intervenção Guiada por Imagem do Hospital Israelita Albert Einstein, São Paulo, SP, Brasil; 3 Departamento de Urologia do Hospital Israelita Albert Einstein, São Paulo, SP, Brasil

**Keywords:** Prostatic Neoplasms, Magnetic, Resonance Imaging, Biopsy, Prostate

## Abstract

**Objective:**

To evaluate the diagnostic efficacy of transrectal ultrasonography (US) biopsy with imaging fusion using multiparametric (mp) magnetic resonance imaging (MRI) in patients with suspicion of prostate cancer (PCa), with an emphasis on clinically significant tumors according to histological criteria.

**Materials and Methods:**

A total of 189 consecutive US/MRI fusion biopsies were performed obtaining systematic and guided samples of suspicious areas on mpMRI using a 3 Tesla magnet without endorectal coil. Clinical significance for prostate cancer was established based on Epstein criteria.

**Results:**

In our casuistic, the average Gleason score was 7 and the average PSA was 5.0ng/mL. Of the 189 patients that received US/MRI biopsies, 110 (58.2%) were positive for PCa. Of those cases, 88 (80%) were clinically significant, accounting for 46.6% of all patients. We divided the MRI findings into 5 Likert scales of probability of having clinically significant PCa. The positivity of US/MRI biopsy for clinically significant PCa was 0%, 17.6% 23.5%, 53.4% and 84.4% for Likert scores 1, 2, 3, 4 and 5, respectively. There was a statistically significant difference in terms of biopsy results between different levels of suspicion on mpMRI and also when biopsy results were divided into groups of clinically non-significant versus clinically significant between different levels of suspicion on mpMRI (p-value <0.05 in both analyzes).

**Conclusion:**

We found that there is a significant difference in cancer detection using US/MRI fusion biopsy between low-probability and intermediate/high probability Likert scores using mpMRI.

## INTRODUCTION

Prostate cancer (PCa) is the most common male malignancy in United States, excluding skin cancers, and the second most common cause of male cancer-related death (1). Diagnosis of PCa increased with the use of prostate-specific agent (PSA) as a blood test for screening. The diagnosis of PCa is made with a histological sample of systematic transrectal ultrasound-guided biopsy (TRUSGB) of the prostate, which is indicated with increased values of PSA blood-test and/or altered digital rectal examination (DRE) (2-4).

PSA, DRE and histological findings are used to determine diagnosis and type of treatment in PCa, however each approach has shortcomings: PSA has low sensitivity (only 36% in patients younger than 60 years old with a PSA value of 2.6ng/mL,) (5, 6), DRE also has low sensitivity (37%) (7), and, when positive, prostate biopsy has up to a 54% underestimation of the Gleason score when compared with prostate specimen (2, 4, 7-12).

PCa screening with a PSA blood test still raises intense debate. The European Randomized Study of Screening for Prostate Cancer (ERSPC) showed a 20% decrease in mortality related to PCa in patients screened with PSA but with considerable rates of overdiagnosis and overtreatment (4.5% of the cases) (3). Others studies have shown rates ranging from 22% to 56% of overdiagnosed PCa (9, 13). Due to this, many cases of indolent and non-aggressive cancers have been discovered and treated, increasing morbidity with impacts on quality of life without changing mortality (2, 9) (14). But overdiagnosis does not have to lead to overtreatment, and active surveillance (AS), in patients with low-risk tumors, can be the modality of choice for patients until early signs of disease progression (1, 3, 4, 13).

Because of the diagnostic limitations of PCa mentioned above, other tools are needed to improve detection, localization and sampling of PCa (7). Advances in 3-Tesla multiparametric (mp) magnetic resonance imaging (MRI) have improved the detection of PCa prior to biopsy (15-18).

The use of mpMRI to guide biopsy has shown to increase the diagnosis of intermediate/high risk PCa and decrease the diagnosis of low-risk tumors (2).

The objective of this study was to evaluate the diagnostic efficacy of mpMRI with different levels of suspicion in detecting PCa, using TRUSGB with US/MRI real-time imaging fusion, with an emphasis on the detection of clinically significant tumors according to histological criteria.

## MATERIALS AND METHODS

This retrospective study was approved by the ethical committee of our institution and a waiver of informed consent was obtained. We performed a database search for patients who received prostate mpMRI for the detection of clinically significant PCa, followed by TRUSG with real-time imaging fusion of US and MRI images, between August 2013 and September 2014. Inclusion criteria were patients who underwent prostate mpMRI and prostate biopsy with US/MRI imaging fusion, both in our institution. Exclusion criteria were: incomplete or poor quality MRI, interval greater than 6 months between MRI and biopsy, and unavailability of histopathological report. Since the aim of our study was to compare the overall mpMRI results with histological analysis based on clinically significant disease, which is classified using all samples together, we also included patients whose additional samples were not identified separately.

All patients underwent mpMRI on a 3 Tesla scanner (Magnetom Trio, Siemens Healthcare, Erlangen, Germany) using a phased-array coil. The mpMRI protocol is described in Table-1.

**Table 1:** mpMRI prostate protocol.

Sequence	Thickness (mm)	Spacing (mm)
T2 FSE axial with fat sat	6	1
T1 GRE axial “in-phase” and “opposed-phase”	6	1
T2 FSE sagittal	3	0.3
T2 FSE axial high resolution	3	0.3
T2 FSE 3D coronal volumetric isotropic	1	-
Diffusion (b50, 400 and 800)	3	0.3
T1 GRE VIBE pre-contrast	3	-
T1 GRE VIBE dynamic post-contrast	3	-
T1 GRE VIBE post-contrast pelvis	4	-

MpMRI images were read independently by two radiologists (in cases of discrepancies a consensus agreement was achieved), and scored using a 5-point Likert scale of probability of having clinically significant PCa, based on the PI-RADS version 1 classification proposed by the European Society of Urogenital Radiology (1-clinically significant disease is very unlikely to be present, 2-clinically significant disease is unlikely to be present, 3-clinically significant disease is equivocal, 4-clinically significant disease is likely to be present and 5-clinically significant disease is highly likely to be present) (16, 17).

In patients indicated for biopsy (PSA level, PSA velocity, mpMRI result, DRE and/or urologist discretion), a 14-core TRUSGB was performed by acquiring up to four fragments of each suspicious area on mpMRI (using T2-weighted, diffusion and/or dynamic contrast-enhanced sequences to fuse the images) and a systematic biopsy in a sextant pattern (12 fragments of the peripheral zone and 2 of the central gland). One of two different ultrasound devices was used to fuse the images and perform the biopsies: Aplio 500 with Smart Fusion (Toshiba Medical System Corporation, Minato, Tokyo, Japan) or LOGIC E9 with imaging fusion software (GE Healthcare, Little Chalfont, United Kingdom). Patients with low probability of PCa on mpMRI (Likert 1 and 2) were submitted to a 14-core TRUSGB only, when clinically indicated.

A certified pathologist evaluated the biopsy specimens. Clinical significance for PCa was established based on Epstein criteria and included any Gleason pattern 4 or higher, or Gleason 3+3 disease with more than 50% of cancer in any core and/or more than 3 positives cores (19).

Statistical analysis was made using the Shapiro-Wilk test to decide between mean (and standard deviation-SD) or median (and first and third interquartile intervals-IQs) for age, PSA levels and prostate weight. To study the association between suspicion on mpMRI and Gleason score we used the Spearman correlation coeffcient, and because the variables had more than two categories, we performed subanalysis with Chi-square partitions. Finally, to calculate the association between suspicion level on mpMRI with PSA level and with Gleason score we used the Kruskal-Wallis test.

## RESULTS

**Figure 1:**
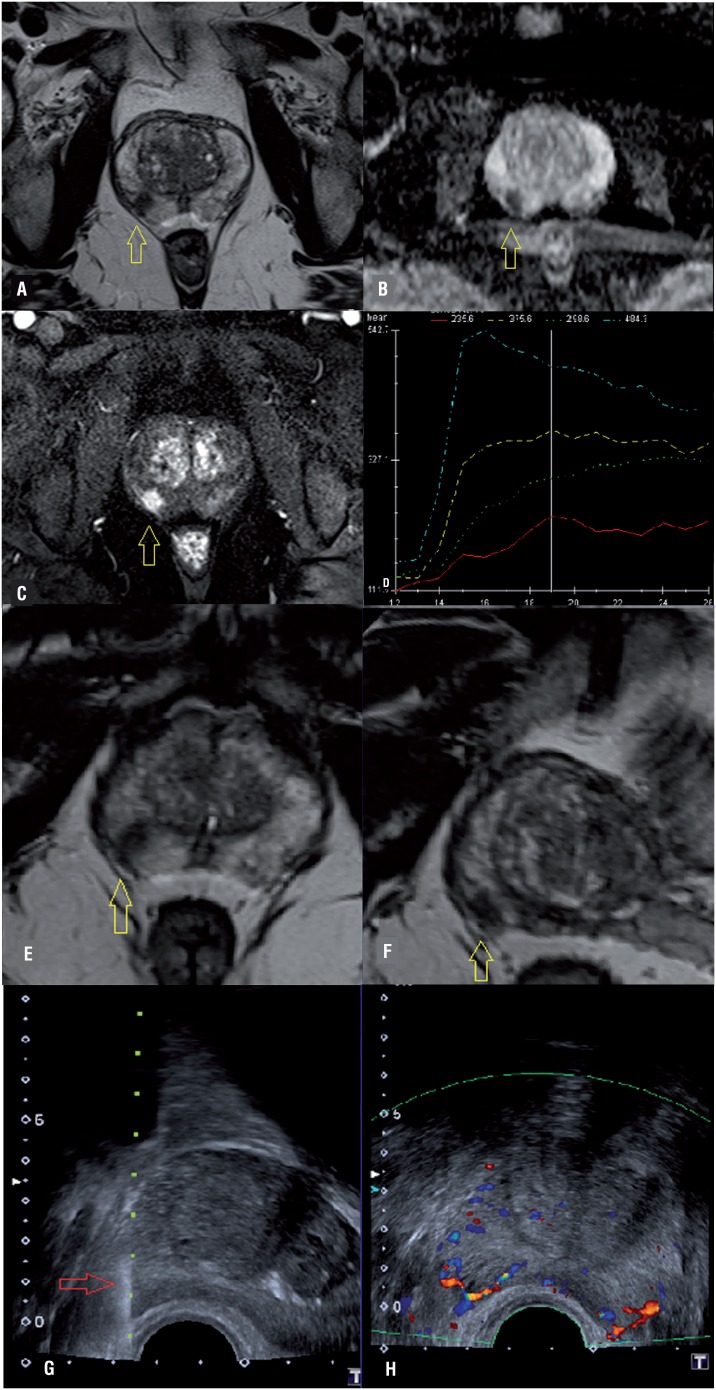
MpMRI example of a suspicion lesion on the right lobe of peripheral zone of the prostate: A) a round lesion on T2 weighted-image; B) ADC map shows diffusion restriction; C) dynamic-contrast-imaging with hypervascularization and D) washout (blue line). Patient received US/MRI-guided biopsy where we can see the lesion on MRI (E and F) and in real time on US (G), helping to target the lesion (H).

A total of 195 consecutive patients were included in the study; six patients were excluded due to an interval greater than 6 months between MRI and biopsy leaving a final casuistic of 189 patients for analysis. The mean age was 58.12 years old (SD±9.16), median serum PSA was 5.0ng/ mL (IQs 3.6-7.1) and median prostate volume was 45cc (IQs 34-62). The mean of additional fragments on suspicious areas was three (range: two to four). Of 189 patients, 153 had never received a prostate biopsy and 36 had received at least one with negative results. MpMRI was considered suspicious for PCa in 103 patients (Likert 4 or 5), equivocal in 68 (Likert 3) and low level of suspicion in 18 (Likert 1 or 2) (Figure-1).

**Figure 2:**
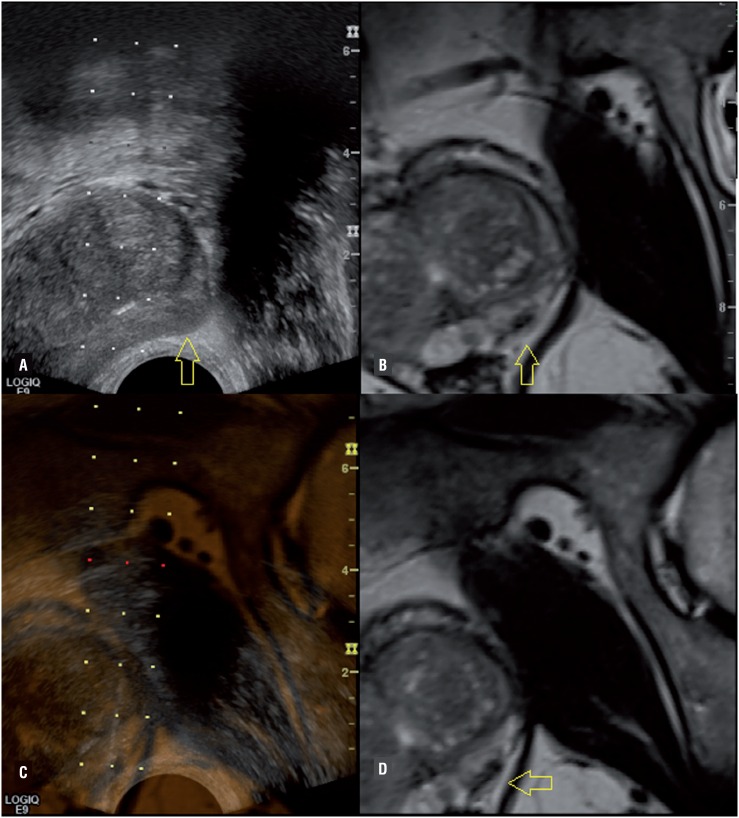
A suspicious lesion on mpMRI (images B and D) submitted to a US-MRI fusion biopsy. The lesion was not seen on US (arrow in A) and the biopsy was performed based on mpMRI (arrow in C). The biopsy result was Gleason 3+4 in all tree fragments of this area.

Of the 189 patients who performed US/MRI biopsies, 110 (58.2%) had positive biopsy for PCa. Of those cases, 88 (80%) were clinically significant, accounting for 46.6% of all patients (Figure-2). The overall distribution of US/MRI biopsy for negative biopsy, positive biopsy with clinically non-significant cancer, and positive biopsy with clinically significant cancer was, respectively, 1/0/0 in Likert 1, 10/4/3 in Likert 2, 42/10/16 in Likert 3, 21/6/31 in Likert 4, and 5/2/38 in Likert 5, resulting in positive indications for clinically significant prostate cancer of 0%, 17.6%, 23.5%, 53.4% and 84.4% in Likert scores 1, 2, 3, 4 and 5, respectively (Table-2).

**Table 2:** Suspicion level on mpMRI and biopsy results.

		Biopsy		
		Negative	Positive non-significant	Positive significant
Suspicion on mpMRI	Very low	1	0	0
	Low	10	4	3
	Equivocal	42	10	16
	Moderate	21	6	31
	High	5	2	38

p-value <0.001

There was a statistically significant difference in the level of suspicion on mpMRI (very low, low and equivocal probability versus intermediate and high probability, or Likert 1, 2 and 3 versus Likert 4 and 5) compared with biopsy results in terms of clinically significant disease (negative biopsy and non-significant positive biopsy versus significant positive biopsy). This was also true when we included the “equivocal” category on mpMRI as positive (Likert 1 and 2 versus Likert 3, 4 and 5) (p<0.001 in both analyses) (Table-3).

**Table 3:** Suspicion level on mpMRI and biopsy results according clinical relevance.

		Biopsy	
		Negative + positive clinically non-significant	Positive clinically-significant
Suspicion on mpMRI	Very low	1	0
	Low	14	3
	Equivocal	52	16
	Moderate	27	31
	High	7	38

p-value <0.001

**Figure 3:**
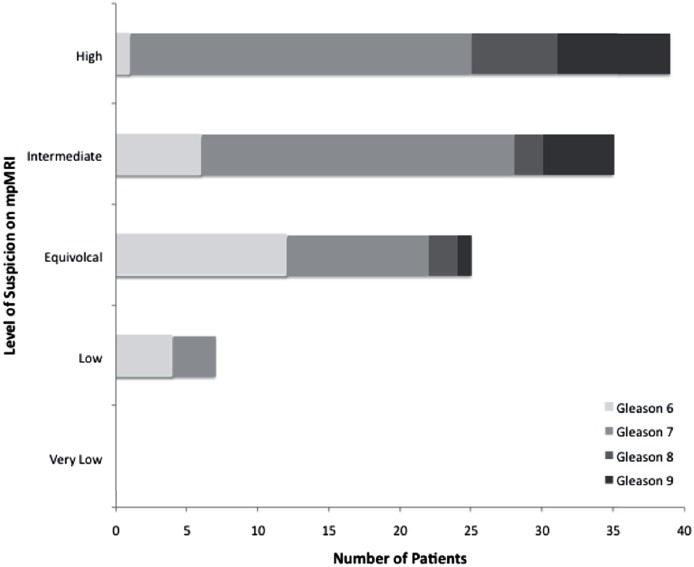
Frequency of patients in each Gleason score value in correlation with level of suspicion on mpMRI (n=110).

We observed that most patients with a Gleason score of 6 were considered to have an equivocal level of suspicion for PCa on mpMRI, while most patients with Gleason scores of 7, 8 and 9 were considered as moderate or high suspicion for PCa, as shown in Figure-2. The association between suspicion on mpMRI and Gleason score was moderately positive, with a coefficient of 0.435 (p<.001) (Figure-3).

In the three positive cases of clinically significant cancer that we classified as Likert 2 (false negative), two had only one positive fragment on biopsy (5% and 10% of Gleason 3+4 and 4+3, respectively), and the third case had 5% of the fragments committed with Gleason 3+4. On the other hand, in the five negative cases of clinically significant cancer that we had classified as Likert 5 (false positive), one lesion seen on MRI was diagnosed as a leiomyoma, two lesions were acute prostatitis, one lesion was chronic prostatitis and one was a small lesion (5mm) in a large prostate (125cc), which, we believe, might have resulted in undersampling during the biopsy.

## DISCUSSION

Our results show that mpMRI, performed on a 3 Tesla scanner without endorectal coil, has the ability to stratify the risk of detection of clinically significant prostate cancer on US/MR fusion biopsy, therefore increasing the likelihood of positivity of the procedure and decreasing unnecessary biopsies.

The prostate is the only solid organ that has the diagnosis of tumor made by non-target sampling biopsy (20). Other studies have shown that mpMRI has the capability to detect suspicious areas for PCa and to target the US-guided biopsy of lesions seen on mpMRI (8, 15-17, 21, 22), and have already suggested higher identification rates than random biopsy (23). It has been shown that mpMRI increases the detection of clinically significant PCa (including those located in the anterior region of the prostate, usually blinded on systematic biopsy), without increasing diagnosis of clinically insignificant disease (8, 18, 24).

Performing mpMRI in patients with clinical-laboratorial profiles suspicious for PCa could prevent unnecessary systematic biopsies and a delay in diagnosis and treatment (7).

Currently, transrectal systematic US-guided biopsy is the modality of choice for prostate biopsy, however it is limited in that it can miss and undersample existing tumor (7, 15, 25). The use of mpMRI prior to biopsy has resulted in the development of new methods to increase the detection of clinically significant PCa: 1) in-bore MRI-guided biopsy, which is expensive and time-consuming; 2) cognitive fusion, where the lesion location is estimated by the operator; 3) US/MRI-fusion-guided biopsy, in which the pre-biopsy mpMRI is fused in real-time by a navigation system, allowing additional sampling of suspicious lesions through direct visualization during the procedure (8, 15, 25-27).

With the aim to make recommendations for conduct, interpretation, and reporting of prostate mpMRI for PCa detection and localization, a European Consensus Meeting was performed and a 5-point scale was suggested to indicate the probability of malignancy (PI-RADS system) (16). We used a 5-point subjective scale of probability (Likert scale) based on the PI-RADS classification, but in which the overall impression of the imaging findings is the most important aspect of grading. There are many mpMRI prostate studies in the literature using Likert, PI-RADS and also comparing both classification methods (28, 29). Rosenkrantz et al. compared the systematic model proposed by the European Consensus (PI -RADS) with the probability score (Likert) and showed that radiologists performed well localizing PCa with both methods, however tumors in the central gland had better correlation with the specimen using the Likert scale (30). PI-RADS is a promising method and used by many radiologists, but its implementation is still a cause for debate. Because of that we used a scoring system that we believe adds value of a standardized method (such as PI-RADS) but also relies on the radiologist's experience and learning curve.

US/MRI-fusion-guided biopsy increases the detection of clinically significant PCa (especially with higher suspicion level on mpMRI) when compared with systematic biopsy, positively impacting treatment decisions and outcomes (8, 20). Thompson et al. correlated the biopsy findings with PI-RADS scores on mpMRI, and found high negative predictive value and moderate positive predictive value for the detection of PCa, demonstrating a potential screening test to guide biopsy decisions (12). Porpiglia et al. showed that mpMRI had better accuracy to diagnose PCa in patients with a negative biopsy than promising biomarkers (PSA3 and p2PSA) (11).

Our results show that 3 Tesla mpMRI without an endorectal coil is a non-invasive technique that helps to detect clinically significant PCa, with high concordance between the probability of clinically significant disease on mpMRI and biopsy results.

It is known that mpMRI has limited sensitivity for the detection of lesions smaller than 5mm (20, 27), possibly explaining the two cases of clinically significant tumors (Gleason score 7) that were classified as low suspicion on MRI and came up with one positive fragment on the biopsy.

Our study has some limitations. It was a retrospective study, which might have introduced some selection bias, but we believe this did not affect the results, since all cases during the period of the study were consecutively included. The biopsy fragments were not identified separately and we did not evaluate the increment value of fusion, but we opted for such methodology because the histological criteria's for clinical significance disease using Epstein's criteria relies on overall result of the samples. MpMRI cases were read independently and a final report was reached by consensus agreement, but interobserver variability was not evaluated. Also, we used the biopsy as reference test, and could miss or misclassify some tumors as compared to the prostatectomy specimen; however, we believe that using a prostatectomy specimen would substantially limit the casuistic, and we would have to consider the effect of a reference standard on our population (biopsy for negative, prostatectomy for positive cases). Finally, because it was a retrospective project with the aim of studying mpMRI performance, we did not perform a follow-up on patients and record the number of complications related to biopsy.

## CONCLUSIONS

In conclusion, we found that mpMRI performed on a 3-Tesla scanner without an endorectal coil and using a Likert scale has significant correlation with biopsy results in terms of cancer detection and clinical significance. This study highlights the potential use of this method in clinical practice to manage patients with clinical suspicion of PCa, decreasing unnecessary biopsies and overdetection of clinically non-significant tumors, and increasing the diagnosis of clinically significant cancers.
